# Exploring antimicrobial resistance determinants in the Neanderthal microbiome

**DOI:** 10.1128/spectrum.02662-23

**Published:** 2024-06-25

**Authors:** Gomathinayagam Sankaranarayanan, Gothandam Kodiveri Muthukaliannan

**Affiliations:** 1 School of Biosciences and Technology, Vellore Institute of Technology, Vellore, Tamilnadu, India; University of Manitoba, Winnipeg, Manitoba, Canada

**Keywords:** Neanderthal microbiome, metagenomics, antimicrobial resistance, ancient DNA, fecal microbiome

## Abstract

This study aimed to investigate the presence of antimicrobial resistance determinants (ARDs) in the Neanderthal microbiome through meticulous analysis of metagenomic data derived directly from dental calculus and fecal sediments across diverse Neanderthal sites in Europe. Employing a targeted locus mapping approach followed by a consensus strategy instead of an assembly-first approach, we aimed to identify and characterize ARDs within these ancient microbial communities. A comprehensive and redundant ARD database was constructed by amalgamating data from various antibiotic resistance gene repositories. Our results highlighted the efficacy of the KMA tool in providing a robust alignment of ancient metagenomic reads to the antibiotic resistance gene database. Notably, the KMA tool identified a limited number of ARDs, with only the 23S ribosomal gene from the dental calculus sample of Neanderthal remains at Goyet Troisieme Caverne exhibiting ancient DNA (aDNA) characteristics. Despite not identifying ARDs with typical ancient DNA damage patterns or negative distance proportions, our findings suggest a nuanced identification of putative antimicrobial resistance determinants in the Neanderthal microbiome’s genetic repertoire based on the taxonomy–habitat correlation. Nevertheless, our findings are limited by factors such as environmental DNA contamination, DNA fragmentation, and cytosine deamination of aDNA. The study underscores the necessity for refined methodologies to unlock the genomic assets of prehistoric populations, fostering a comprehensive understanding of the intricate dynamics shaping the microbial landscape across history.

**IMPORTANCE:**

The results of our analysis demonstrate the challenges in identifying determinants of antibiotic resistance within the endogenous microbiome of Neanderthals. Despite the comprehensive investigation of multiple studies and the utilization of advanced analytical techniques, the detection of antibiotic resistance determinants in the ancient microbial communities proved to be particularly difficult. However, our analysis did reveal the presence of some authentic ancient conservative genes, indicating the preservation of certain genetic elements over time. These findings raise intriguing questions about the factors influencing the presence or absence of antibiotic resistance in ancient microbial communities. It could be speculated that the spread of current antibiotic resistance, which has reached alarming levels in modern times, is primarily driven by anthropogenic factors such as the widespread use and misuse of antibiotics in medical and agricultural practices.

## INTRODUCTION

Antibiotic resistance has existed in nature long before antibiotics were introduced for clinical use. Antibiotic resistance genes have been found in metagenomes retrieved from ancient ice cores (1). Microbes in natural settings are known to exist in both competitive and symbiotic environments. Antimicrobials and antimicrobial resistance are, thus, inevitable factors in aiding microbes to maintain their population in their respective niches. Simultaneously, these niches provide fertile ground for the emergence of resistance in nearby susceptible organisms through horizontal gene transfer since exposure to antimicrobials initiates the spread of antibiotic resistance genes to susceptible organisms (2, 3).

It is believed that ancient civilizations, such as the Egyptians, used natural substances like mud, moldy bread, and saliva for disinfection purposes, even though they had no knowledge of the existence of microbes (4, 5). Much earlier than these human civilizations, recent studies have uncovered evidence of poplar containing salicylic acid and traces of *Penicillium* genomic sequences in the dental calculus of a Neanderthal individual with a severe dental abscess (6). It could be speculated that, to treat microbial infection, antimicrobials had been used inadvertently by Neanderthals, thus creating a conducive environment for antimicrobial resistance genes to concentrate in these niches. Neanderthals, an extinct species of hominid, inhabited diverse regions of Europe and Asia from approximately 200,000 to 40,000 years ago (7). They left a substantial archeological record in the caves they had occupied. In recent years, the analysis of ancient metagenome DNA (aDNA) isolated from the dental calculus and coprolites of ancient beings has yielded fresh insights into their lives, shedding light on aspects such as their diet, health, and relationships with other hominid species.

One of the well-studied Neanderthal sites is El Sidron, a cave system located in northern Spain. This site has yielded numerous well-preserved Neanderthal fossils, as well as artifacts and other archeological materials. Studies of the dental calculus from these fossils have revealed information about Neanderthal diet and their interactions with microbes in their environment (7, 8). Another important site is Spy Cave, located in Belgium, which contains two adult Neanderthal skeletons, as well as numerous artifacts and other remains, paving the way for a quality archeological study of these ancient beings (9). The study of aDNA from this fossil dental calculus has provided new insights into the relationships pertaining to dietary habits between Neanderthals and other hominid species (7).

El Salt is another site in Spain that has yielded important Neanderthal fecal materials in coprolite form, which helped investigators understand the gut microbiome and diet of Neanderthals. Fumane Cave, located at the foot of the Alps in Italy, is another important site that has added more information about Neanderthals. This cave contains evidence of Neanderthal occupation between 47.6 ka and 41.9 ka (thousands of years ago) cal BP, including morphometric data of dental remains of Neanderthals.

This study aims to explore the presence of antimicrobial resistance determinants in the microbiome of Neanderthal remains such as coprolites and dental calculus. To achieve this, we retrieved the metagenome data from the public genomic repositories originally collected from studying dental calculus and fecal sediments at various Neanderthal sites across Europe, including El Sidron, Spy Cave, El Salt, Pesturina, Goyet Troisieme Caverne, and Fumane Cave (10). We chose to construct genes of potential antimicrobial resistance determinants directly from the metagenome sequence reads instead of first assembling the reads into contigs to avoid overlooking low-coverage regions by assembly programs.

However, the study of aDNA presents additional challenges, including DNA fragmentation and base misincorporation during sequencing due to cytosine deamination. To address these issues, we have employed specific procedures to accurately identify the presence of AMR genes in ancient metagenome raw reads. Our findings may hold crucial implications for comprehending the presence of antimicrobial resistance in Neanderthal microbiomes and the impact it might have had on their health.

## RESULTS

### Read fragmentation in ancient DNA and putative ancient ARDs

**Fig 1:**
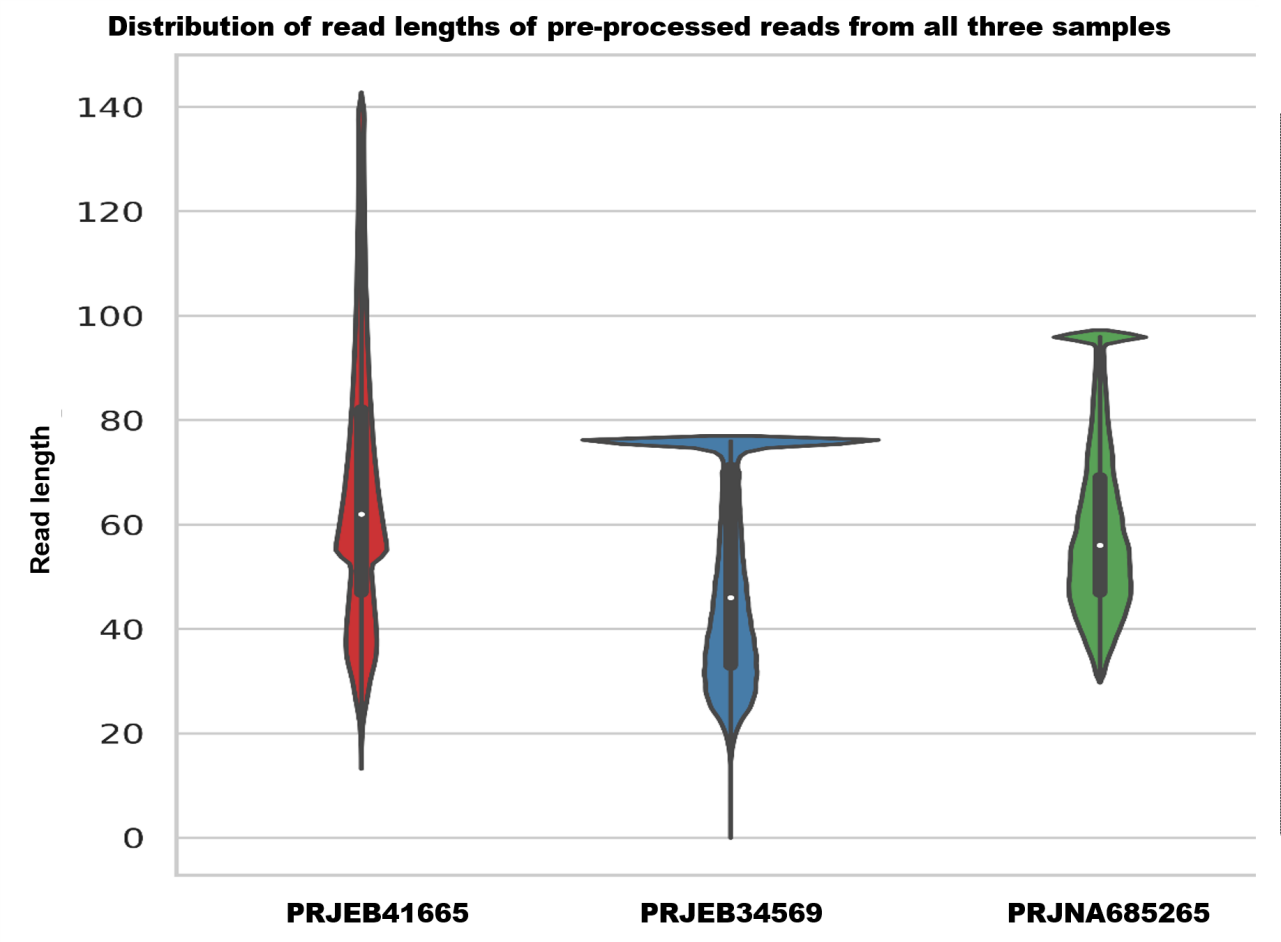
Read length distributions of various samples in this analysis showing varying read fragmentation levels.

Read fragmentation is a common challenge encountered in studies on aDNA. In our analysis, we observed that the reads were fragmented in all three samples after adapter removal. In the El Salt fecal sediment project (PRJEB41665) (10), the reads’ size ranged from 15 bp to 140 bp, which is consistent with previous studies that have reported similar read sizes in aDNA samples. The second study (PRJEB34569) had read sizes in the range of 1 bp to 75 bp (11), which is notably shorter than the other two samples, likely due to the greater degree of degradation of DNA in the sample. Despite this fragmentation, we were able to identify putative ancient antimicrobial resistance determinants in certain samples in the second study. The third study (PRJNA685265) had read fragment size ranging from 30 bp to 95 bp (6), as shown in Fig. 1. It is worth noting that the sizes of reads mapped to the ARD database were also fragmented, and it was in the range of 30 to 77 bp.

### Ancient sequence read mapping with KMA and GROOT

From the El Salt site (PRJEB41665), 14 coprolite samples had metagenome data, yielding 7.611 Gbp (billion base pairs) and 1,532 reads that were mapped to the ARD (antimicrobial resistance determinants) database by KMA aligner (12). Sixteen dental calculus (several sites across Europe) samples from project PRJEB34569 had 43.1 Gbps data, from which 181,139 reads were mapped to the ARD database. Special emphasis was placed on the antibiotic inactivation resistance mechanism over other resistance mechanisms. Out of 117 hits, 24 were related to the antibiotic inactivation type (Table S2 in the repository https://zenodo.org/doi/10.5281/zenodo.10532261). Another four dental calculus samples from El Sidron and Spy Cave (PRJNA685265) yielded 24.7 Gbp, from which 4,253 reads were mapped to the ARD database. None of those mapped reads were for antibiotic inactivation resistance type, while nine were mapped to other antibiotic resistance mechanisms. The dental calculus samples from PRJEB34569 had the highest sequencing throughput and count of mapped ARD reads (Table 1). The results showed that in the GROOT alignment method (13), the reads from all three studies did not cover more than 40 percent of any antibiotic resistance genes in the redundant database (Table S1 in the repository https://zenodo.org/doi/10.5281/zenodo.10532261). However, reads aligned using the KMA tool were able to identify antibiotic resistance determinants (ARDs) in the metagenome sequences of the second study with considerable coverage for several antibiotic resistance genes in the database. This suggests that the KMA tool was able to provide a more comprehensive alignment of the ancient DNA sequenced reads to the antibiotic resistance gene database compared to GROOT, despite fragmentation and base pair substitutions. It is possible that the difference in coverage could be due to the different algorithms used by the two tools during the alignment process. Overall, these preliminary results suggest that presently, the KMA tool may be more effective for identifying ARDs in ancient metagenomic samples. However, further validation was necessary to confirm these findings, and it is also important to ensure that higher recovery in KMA alignments is not due to ambiguous alignments or misalignments.

**TABLE 1:** Summary of results from ARD identification through KMA aligner

Study	No. of samples	Sequence throughput in base pairs	Reads mapping to the ARD database	Count of hits—antibiotic inactivation vs others
Coprolite	14	7.611 Gbps	1,532	0/13
Dental calculus (others)	16	43.1 Gbps	181,139	24/117
Dental calculus (El Sidron and Spy cave)	4	24.7 Gbps	4,253	0/9

**Fig 2:**
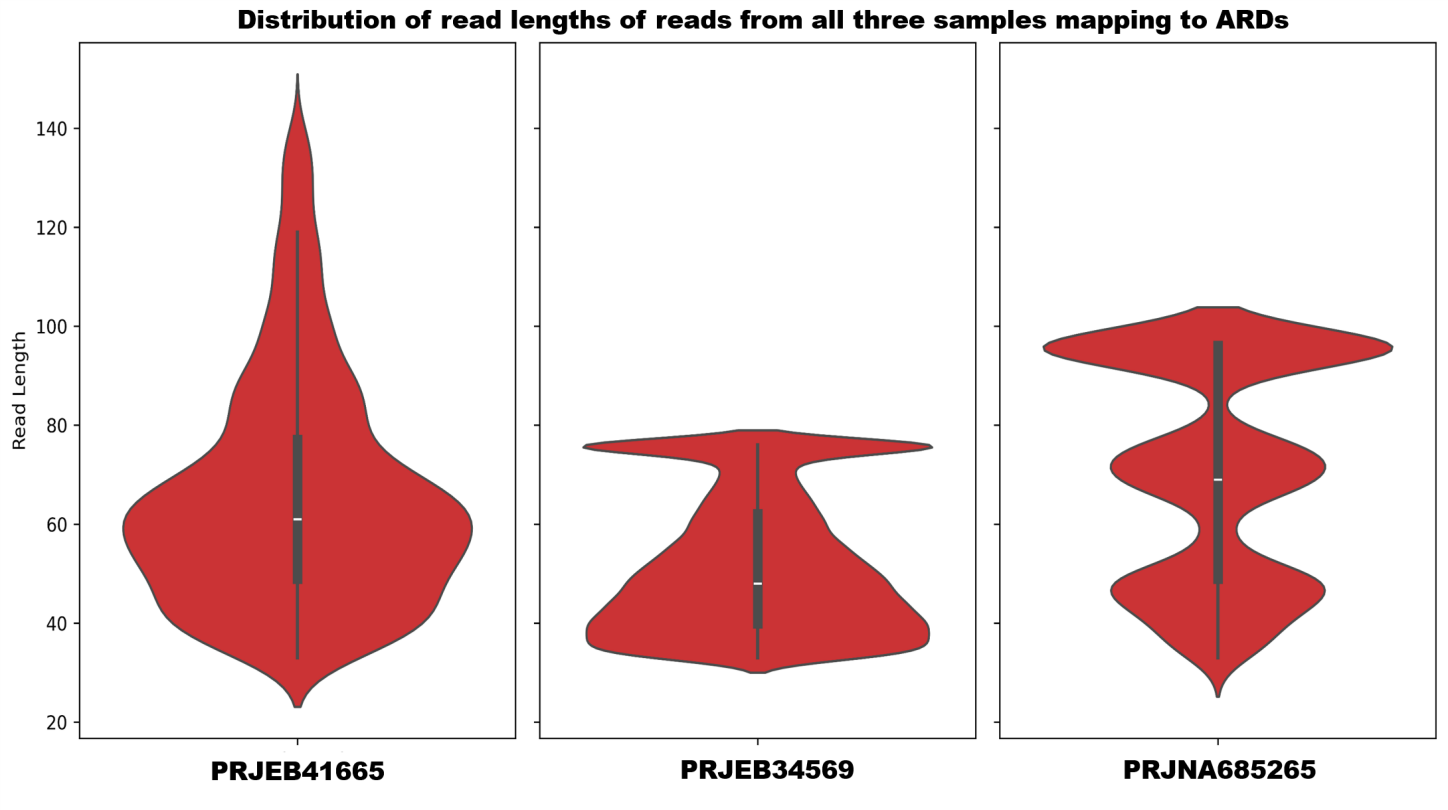
Distribution of read lengths based on reads mapping to ARDs in the database.

The reads mapping to the ARDs (including antibiotic inactivation class) by KMA aligner were also fragmented, as shown in Fig. 2.

### Validation of KMA to detect ARDs in ancient metagenome reads

To validate KMA, ancient reads were simulated from 30 contemporary colistin resistance genes and spiked into one of the ancient metagenome data. KMA successfully identified 10 out of the 30 spiked genes, achieving a breadth of coverage above 70% and a depth of coverage of 3%. Conversely, Burrows–Wheeler Aligner (BWA) identified 393 out of the 30 spiked genes using the same coverage thresholds but with a mapping quality of 0 (14). (Table S1 in the repository https://zenodo.org/doi/10.5281/zenodo.10532261)

This discrepancy in BWA’s performance is due to its limitation in handling reference sequences with considerable similarity between them. This is the reason that BWA is not extensively employed in detecting antibiotic resistance genes (ARGs) within ARG detection tools. In contrast, KMA is utilized in tools like ResFinder and others developed by the Center for Genomic Epidemiology, DTU (15).

Despite KMA’s effectiveness, it could not recover all 30 ARDs from the metagenome reads due to the DNA damages introduced by using Gargammel (16). To further validate KMA’s effectiveness in real-world data, we took samples from less-numerous less-ancient data and also contemporary humans’ dental calculus metagenome reads. In the investigation of dental calculus samples from both the pre-antibiotic era (PRJEB34569 and PRJEB35483) and contemporary humans (PRJNA685265), the research aimed to validate the capability of KMA in detecting antibiotic resistance genes across diverse temporal contexts Table S1 in the repository https://zenodo.org/doi/10.5281/zenodo.10532261). The pre-antibiotic era samples exhibited a notable abundance of macrolide (MLS23S), aminoglycoside (A16S, RRS), and tetracycline (TET16S) resistance genes, alongside sporadic occurrences of vancomycin (*van*), fosfomycin (*fos*), and rifampin (RPOB) resistance genes. As anticipated, the contemporary dental calculus samples displayed higher abundance of antibiotic resistance genes, with sporadic instances of tetracycline (*tet*M), β-lactam (*bla*a), erythromycin (*erm*), and aminoglycoside (*aac*6) resistance genes. Other identified resistance genes included ribosomal protection proteins, efflux pumps, and DNA topoisomerases (GYRA, GYRB, and GYRC) (Table S1 in the repository https://zenodo.org/doi/10.5281/zenodo.10532261). Notably, certain resistance genes, including *fos*, CDSA, *van*, *ant*, and *cat*, were found relatively less across the samples. The frequent occurrence of these resistance genes across various samples implies their widespread and abundant presence, validating KMA’s effectiveness in detecting ARGs from both ancient and contemporary dental calculus metagenomes.

### Environmental contamination assessment by source tracking of taxonomy

Among the identified ARDs, a significant number comprised 23s or 16s sequences. Due to their recovery from ancient reads, pinpointing point mutations for confident identification as determinants of antibiotic resistance is challenging. Nevertheless, an attempt was made to enhance the taxonomic understanding by searching these genes against the NCBI-BLAST database (17). This study aimed to infer the likely source of these sequences based on the identified taxa. In such an examination of microbial sources within the ancient metagenome sample, a broad categorization across airborne, soil sediment, and other origins has been conducted. Notably, airborne sources were discerned through dental calculus, with two species identified (*Rahnella aquatilis* and *Propionibacterium australiense*) and a singular species (*Pseudonocardia saturnea*) in coprolite. Conversely, El Sidron yielded no airborne species. Look up for soilborne bacterial contamination revealed one species (*Acinetobacter tandoii*) in dental calculus, two species (*Amycolatopsis acididurans* and *Halocatena marina*) in coprolite, and one species (*Mycobacteroides abscessus*) in dental calculus from El Sidron. Overall, airborne sources were limited (three in total), while soil sediment sources exhibited a marginal presence (four in total). Predominantly, non-airborne and non-soil sediment sources, particularly associated with oral and gastrointestinal domains, were identified, exemplified by 18 species in dental calculus samples from the second study (various sites in Europe) and seven species in the third study (El Sidron). This implies environmental contamination on the ancient metagenome sample has been limited to an extent.

### Source characterization by taxonomy search of antibiotic inactivation type ARDs

The data set analysis reveals a diverse array of AMR genes of antibiotic inactivation mechanism, with notable occurrences of OXA-211, OXA-48, and OXA-274 beta-lactamase families, Class A and C beta-lactamases, aminoglycoside resistance genes (ANT(3’’)-II, *aac*6), as well as tetracycline (*tet*M) and macrolide (*erm*) resistance genes. Taxonomically, *Acinetobacter species*, including *A. johnsonii* and *A. guillouiae*, dominate, alongside *Streptococcus*, *Enterobacter*, *Shewanella seohaensis*, *Corynebacterium singulare*, and *Klebsiella pneumoniae*. Functional domain analysis through InterproScan highlights crucial domains associated with antibiotic resistance, including penicillin-binding proteins, transpeptidase, adenine methyltransferase, acyltransferase (GNAT), erm, and translational GTP-binding domains (18). Given the common association of *Acinetobacter* with environmental sources, particularly soil and water, their presence raises concerns about the influence of environmental contamination on the detected ARDs with antibiotic inactivation resistance type. In contrast to the abundant presence of *Acinetobacter* species, the occurrence of taxa such as *Streptococcus, Enterobacter, Shewanella seohaensis, Corynebacterium singulare,* and *Klebsiella pneumoniae*, commonly associated with oral and gastrointestinal environments, was discerned. However, their numbers were comparatively fewer than those of the former.

### DNA damage in the reads mapping to ARDs

**Fig 3:**
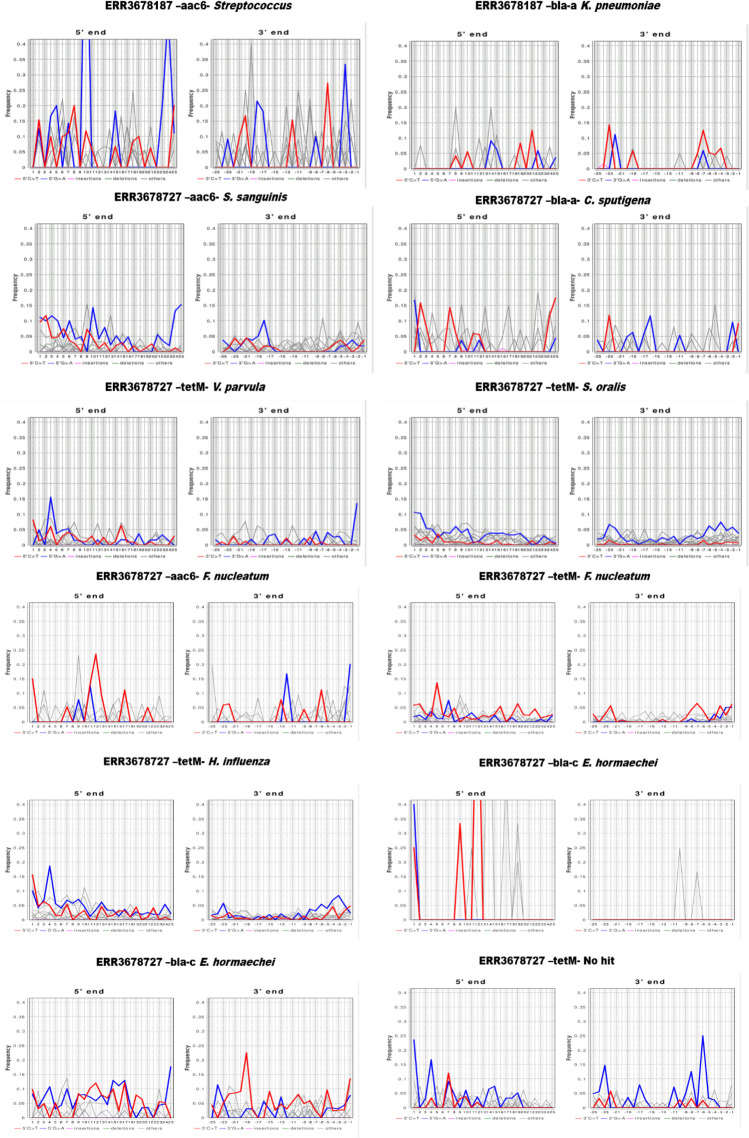
The DNA damage pattern of reads mapping to ARDs after mapDamage2 analysis.

After read fragmentation evaluation, we assessed the DNA damage in the reads mapping to ARDs using the mapDamage2 software (19). Specifically, we looked for C to T deamination in the 5' end and G to A substitution in the 3' end (complimentary) of the reads. Our analysis showed that only the reads mapping to the 23s subunit protein and a putative peptidoglycan transpeptidase in the Goyet Troisieme Caverne samples had DNA damage patterns typical of aDNA fragments (Table S1 in the repository https://zenodo.org/doi/10.5281/zenodo.10532261). It is worth noting that these three ARDs can confer resistance only if they have specific mutations. In contrast, the reads mapping to antibiotic inactivation resistance type or enzymatic antibiotic resistance proteins such as beta-lactamases, flavin-dependent monoxygenases, and multidrug efflux proteins such as MATE did not show sufficient DNA damage patterns, as shown in Fig. 3.

**Fig 4:**
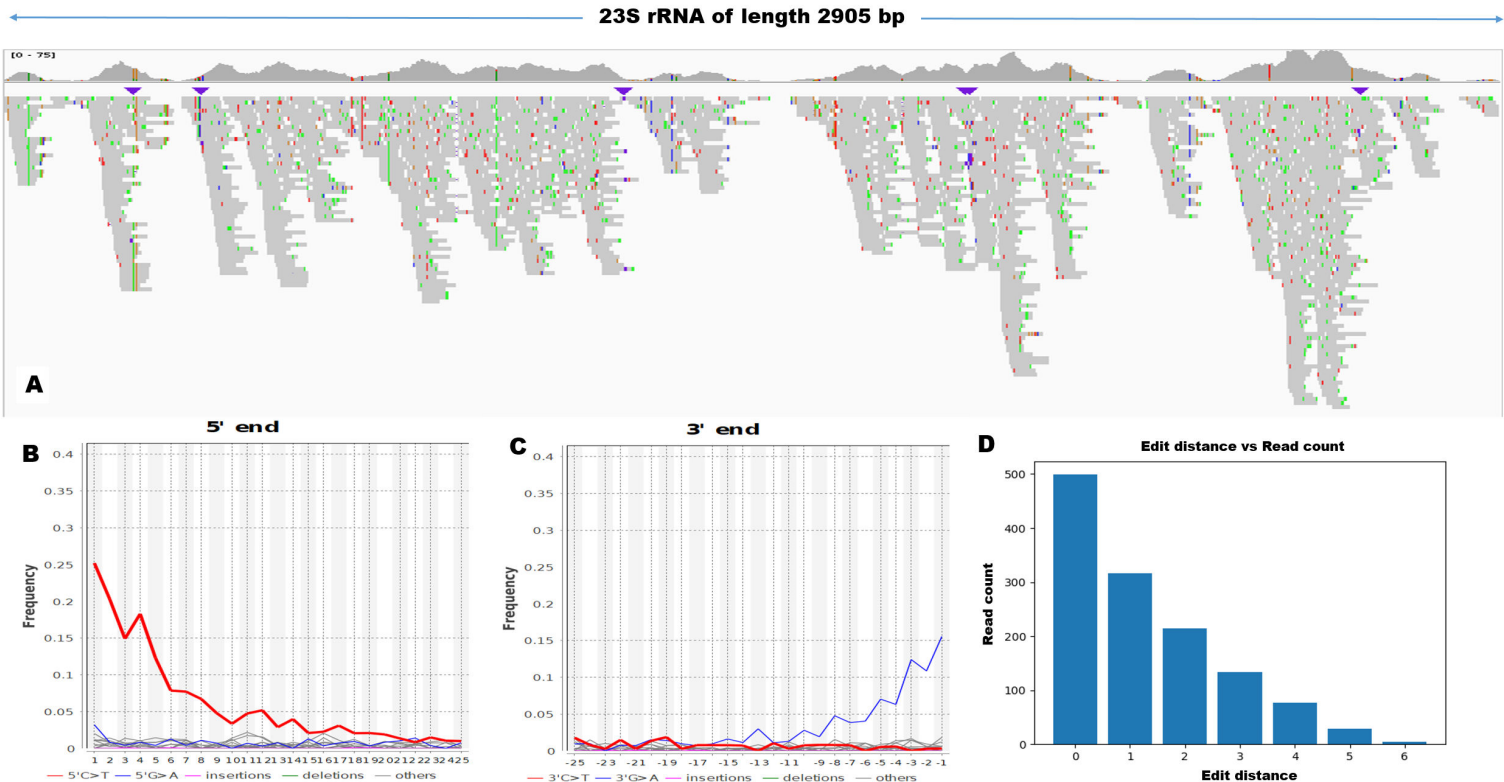
(A) Visualization of BAM alignment in IGV of reads mapping to gene 23s rRNA from Goyet Troisieme Caverne. Purple downward arrows indicate insertions in reads with respect to the reference. (B and C) are DNA damage patterns of reads from 5’ ends and 3’ ends, respectively. (D) is the plot showing perfectly declining edit distance distribution of genes, which has a negative distance proportion of 1.

To confirm the specificity of the reads mapping to ARDs, we used the negative distance proportion method described by Hubler *et al*., 2019 (20). We analyzed the read distribution based on the edit distance of reads from the reference gene. Specifically, the reads distributed based on edit distance should have a negative distance proportion between 0.9 and 1 to assume that the reads are specific to that ARD. Though 11 out of all the ARDs had a negative distance proportion comfortably between the set range, indicating specificity, only the reads mapping to the 23s subunit protein had the authentic ancient DNA damage pattern (Fig. 4B and C). Therefore, it was concluded that only the reads mapping to one of the 23s subunit proteins could be considered endogenous reads of ancient origin according to the HOPS method, while all the other 10 genes could not be classified as such.

## DISCUSSION

In this study, we aimed to explore the presence of antibiotic resistance determinants (ARDs) in the Neanderthal microbiome directly from metagenomic data. Our analysis pipeline included compensating for the effects of control samples, assessing DNA damage patterns in reads mapping to ARDs, and assessing the pattern of edit distance distribution to increase the confidence of reads as endogenous. However, we did not identify the ARDs using a *de novo* gene construction approach. Instead, we adopted a targeted locus retrieval approach by mapping the ancient reads to a known set of AMR genes. The primary reasons are that the ancient reads are fragmented, and efficient assembly may not be feasible. Another consideration is that the adapted method provides control over recovering low-represented sequences that might otherwise be overlooked by assembly algorithms. There are a few other targeted locus assemblers, such as SAUTE and aTRAM (21, 22), but they are either computationally resource-demanding or not well-suited for ancient reads. The ancient reads are anticipated to be fragmented due to cytosine deamination at the ends of DNA, leading to “T” nucleotide base substitutions in the reads during sequencing with “As” in the complementary strand. Therefore, it is necessary to reduce the stringency of read alignment programs to effectively map ancient reads, allowing for mismatches due to the aforementioned reasons. After mapping, the specificity of the read alignments had to be cross-verified by additional methods to avoid spurious representation. We hypothesized that, informed by observations of aDNA analysis methods in the literature, the credibility of the endogeneity of the ancient reads is dependent on the following characteristics: (1) fragmentation of the raw reads after removal of adapter sequences, (2) C to T misincorporation and G to A misincorporation due to C-deamination at the ends of the reads, and (3) the pattern of distribution of reads based on edit distance as adapted in the HOPS tool (20).

The DNA fragmentation and deamination of bases could be the possible reasons why GROOT could not produce sufficient alignment with the required coverage level. Also, the GROOT algorithm is not specifically tuned to align ancient reads such as those from the Neanderthal microbiome. This is despite GROOT using a variation graph of modern ARGs instead of a linear reference. Variation graphs take into account the inherent divergence between modern ARGs, thereby improving the chances of mapping reads that are divergent from the representative sequences. It is worth noting that the sequence coverage did not exceed 40, which coincides with the window size provided during the reference indexing step. However, KMA was able to map ancient reads to a few of the ARDs in the database. To the KMA mapping results, to avoid stochasticism, we imposed minimum threshold breadth of coverage to 70% and minimum depth of coverage to 3 before the authentication analysis downstream.

We also used the mapDamage2 tool to assess DNA damage patterns in the reads, which can be indicative of ancient DNA fragments. Both the preprocessed reads and the reads mapping to the ARDs were fragmented. We evaluated the DNA damage pattern in the mapping reads using mapDamage2. However, only reads mapping to specific genes, such as the 23s subunit protein in the metagenome sequenced from the Goyet Troisieme caverne sample, exhibited authentic aDNA damage patterns. None of the reads mapping to enzymatic antibiotic resistance proteins, such as beta-lactamases, flavin-dependent monoxygenases, or multidrug efflux proteins, displayed sufficient DNA damage patterns typical of reads resulting from ancient DNA. Upon further scrutiny using the negative edit distance proposition, only the aforementioned 23s subunit protein gene exhibited an NDP value within the range of 0.9–1.

Apart from the above analyses, a taxonomy search for each sequence of ARDs from the study PRJEB34569, corresponding to antibiotic inactivation mechanisms, was performed using BLAST against the non-redundant nucleotide database. Hits of *Acinetobacter johnsonii, A. ursingii,* and *A. guillouiae,* which are not commonly found in the oral microflora but typically have environmental habitats, were observed. Additionally, the presence of genera like *Klebsiella* and *Enterobacter*, less typical for the oral microbiome but more commonly associated with the gastrointestinal tract, was identified. Based on this, we consider that these ARDs are not likely from the endogenous Neanderthal oral microbiome but from environmental contaminants. However, the taxonomy analysis revealed hits such as *Streptococcus* sp. FDA-ARGOS, *S. sanguinis*, *S. oralis* subs. Dentisani, and genera like *Capnocytophaga*, *Veitonella*, *Fusobacterium*, and *Haemophilus*, all of which are associated with the oral microflora. Consequently, these putative ARDs could be considered originating from the Neanderthal oral cavity. It is worth recalling that none of these hits exhibited a typical aDNA damage pattern or a substantial negative distance proposition.

A previous study found that the reconstructed genome of oral pathogen *Tannerella forsythia* had a gap of 48 kb, which corresponds to a complete tetracycline resistance gene operon in its contemporary counterpart (23). Based on this observation, one may speculate that the current level of antibiotic resistance emergence is entirely anthropogenic. Alternatively, the sequencing depth may not have been sufficient to detect low-abundance ARDs. Nevertheless, it is important to remember that the absence of evidence is not evidence of absence. Antimicrobial resistance is and was always there among microbial communities.

Our approach to identify ARDs in the Neanderthal microbiome however has certain limitations. We set a threshold of at least 70% breadth of coverage for hits from KMA, which may be less stringent for aDNA genomic reads. However, reducing the coverage threshold could potentially introduce false positives as hits. Another limitation is that ARDs may have diverged significantly from modern counterparts, making them undetectable using current databases. A potential method to overcome this limitation could involve remote homology detection of translated sequences. However, given the level of fragmentation, six-frame translation of fragmented nucleotide sequences might also introduce false positives. Furthermore, another limitation is that potential contamination from environmental DNA could confound the identification of endogenous genes in such aDNA samples.

In conclusion, while we did not find any ARDs with typical aDNA damage pattern and negative distance proportion, our study highlights the possible presence of AMR genes based on the taxonomy–habitat correlation in the microbiomes of Neanderthals who once were thought to have used salicylic acid and *Penicllium* molds to treat dental caries. Furthermore, our study emphasizes the importance of enhanced analysis pipelines and improved sequencing procedures to unravel genomic information from pre-history. Such advancements may contribute to a better understanding of the evolution of antimicrobial resistance both before and after the clinical introduction of antibiotics.

## MATERIALS AND METHODS

### Data retrieval and pre-processing

To investigate the Neanderthal microbiome, a search was conducted in the NCBI BioProject database using the keywords “Neanderthal” and “Microbiome.” This search yielded three projects: one with metagenome data from fecal sediments of a Neanderthal settlement in El Salt and other two with metagenome reads of dental calculus from Neanderthal remains from various parts of Europe. To ensure the data set was as comprehensive as possible, additional searches were conducted in two other repositories. The SPAAM community’s AncientMetagenomeDir, a repository of ancient metagenome projects, was searched for Neanderthal metagenome data (24). Raw read data from these three studies were downloaded from the public repositories to obtain a comprehensive Neanderthal microbiome data set. The first study, PRJEB41665, involved the analysis of the microbiome in Neanderthal fecal sediment samples from El Salt. Raw read data from this study were obtained from the European Nucleotide Archive (ENA) (PRJEB41665). Similarly, raw read data from the second study, with NCBI BioProject number PRJEB34569, which analyzed the microbiome in Neanderthal dental calculus samples from several archeological places in Europe, were obtained from the NCBI Sequence Read Archive (SRA) along with reads of experimental and library negative controls. The third study, accessible with the accession number PRJNA685265, contained raw metagenome reads of dental calculus microbiome samples from Neanderthal remains in El Sidron and Spy Cave in Europe.

The downloaded raw reads were quality-checked using FastQC (v0.11.9) (25), and adapters were removed using Fastp (v0.20.1) (26). The quality of each individual file was also checked using FastQC. All the FastQC HTML files were then concatenated into a single file using MultiQC (v1.9) for collective assessment of all raw reads (27).

### Creation of a redundant database of antibiotic resistance genes

To create a redundant database of antibiotic resistance genes, publicly available databases were combined. The following databases were utilized: Comprehensive Antibiotic Resistance Database (CARD) ARG database (v3.0.9) (28), MarillynR Tetracycline Database (http://faculty.washington.edu/marilynr/, accessed in November 2022), MEGARes database (v3) (29), NCBI Refgene Catalog for Antibiotic Resistance Genes (v2020.10.12), ResFinder (v4.1.0) (15), Beta-Lactamase DataBase (http://bldb.eu, accessed in November 2022), NDARO (PRJNA313047, accessed in January 2023), CBMAR (http://proteininformatics.org/mkumar/lactamasedb/index.html, accessed in November 2022), MUSTARD (http://mgps.eu/Mustard/, accessed in November 2022), and ResFinder FG 2.0. It should be noted that MUSTARD and ResFinder FG2.0 are putative antibiotic resistance determinants retrieved from metagenome-derived contigs.

### Processing of the downloaded database

The aforementioned prepared redundant database of antibiotic resistance genes underwent the following processing steps. Initially, the reference sequences were clustered using the Vsearch cluster algorithm (v2.13.4) with a 90% sequence identity threshold (30). The resulting clusters were rendered as a multiple sequence alignment (MSA) format. This MSA of the antibiotic resistance genes was then indexed using GROOT (v1.1.2) software. During the reference indexing step, a sliding window of size 40 was used through the argument “-w” to account for the “optimal” read length of the raw reads. This procedure generated a fingerprint for each graph traversal of the reference sequences, and the corresponding MinHash signature was stored in the Locality sensitive hashing (LSH) forest data structure format. The generated index file was used to align all processed metagenomic raw reads, and each alignment was converted to a BAM file.

In addition, the same redundant database was indexed separately using the KMA tool (v1.3.6) with a k-mer size of 25. The resulting KMA index was utilized to align all processed metagenomic raw reads using the KMA software. The resulting alignments were rendered as a SAM formatted file for each sample with the following parameters “-sam 4 -cge -eq 20.”

### Ancient DNA damage assessment

The SAM/BAM files generated from the GROOT and KMA alignment steps were analyzed for read coverage of antibiotic resistance genes using “samtools coverage” (31). Only genes with a breadth of coverage of 70% or more and an average minimum depth of coverage of 3 were selected for further analysis. Next, the reference genes covered for 70% or more by the reads mapping were split from the bam file using the “bamtools split” utility, and MD tags were added to these nascent bam files using the “samtools calmd” function.

Subsequently, the same BAM files were utilized to assess the extent of DNA damage using mapDamage2. This step yielded vital insights into the degree of DNA damage present in the samples, a critical factor in determining the antiquity of the mapped reads. Additionally, the DNA damage plots were generated using the DamageProfiler tool (32).

Another crucial factor considered was the proportion of mapped reads with a negative distance proportion score exceeding 0.9. The edit distance plots and the negative distance proportion were computed using a custom Python script created in-house.

### Taxonomy assignment of mapped reads

Consensus sequences were derived from the previously individually split BAM files using the “samtools consensus” utility. Taxonomic classification of these consensus sequences was performed using NCBI BLAST, with a set threshold of 60% for coverage and percentage identity. Among these consensus sequences, those identified as potential ARDs which confer resistance by “antibiotic inactivation” were subsequently analyzed using the CARD-RGI’s BLAST tool.

### Retrieval and analysis of relatively less-ancient metagenome data

In addition to Neanderthals, the Bioprojects PRJEB34569 and PRJNA685265 included metagenomes isolated and sequenced from dental calculus samples obtained from relatively less-ancient humans. Additionally, we obtained raw sequence data from Bioproject PRJEB35483, Accessed on: July 2023), which contained a smaller data set from humans dating back approximately 100 years. Furthermore, we retrieved metagenome data from dental calculus samples of modern humans from PRJEB34569. A comprehensive list of the SRA accessions for each Bioproject can be found in Table S1 in the repository https://zenodo.org/doi/10.5281/zenodo.10532261.

### Validation of KMA to detect ancient ARDs using simulated reads

To validate KMA’s capability to detect ancient antibiotic resistance determinants (ARDs), a read simulation approach was employed. Ancient reads were simulated using the Gargammel tool (v1.1.4),(16) utilizing colistin resistance sequences sourced from the ResFinder database (v4.1.1)(15). These simulated ancient reads were then introduced as contaminants into reads generated from metagenome-assembled data from raw reads accessed from ERR4903908, which had been assembled using MEGAHIT (v1.2.9)(33). This combination was established in a 40/60 proportion, with the goal of assessing KMA’s effectiveness in identifying ARDs within ancient reads. These simulated data were pre-processed to remove adapters.

## Data Availability

Supplementary data, created redundant database of ARDs and codes used in this study are available in the public repository here at https://github.com/GomathiNayagam/Delving_into_Neanderthal_microbiome, and the archived version of the same is available at https://zenodo.org/doi/10.5281/zenodo.10532261.
